# Exploring physiological beta-hydroxybutyrate level in children treated with the classical ketogenic diet for drug-resistant epilepsy

**DOI:** 10.1186/s42494-024-00199-8

**Published:** 2025-02-07

**Authors:** Xiaoying Qiao, Zimeng Ye, Jialun Wen, Sufang Lin, Dezhi Cao, Li Chen, Dongfang Zou, Huafang Zou, Man Zhang, Zhibin Chen, Patrick Kwan, Ingrid E. Scheffer, Jiong Qin, Jianxiang Liao

**Affiliations:** 1 Department of Neurology, Shenzhen Children’s Hospital, Shenzhen, 518038 Guangdong China; 2 Department of Medicine (Austin Hospital), University of Melbourne, Melbourne, Victoria 3084 Australia; 3 Department of Neurosciences, Central Clinical School, Alfred Hospital, Monash University, Melbourne, Victoria 3004 Australia; 4 Department of Medicine, Royal Melbourne Hospital, The University of Melbourne, Parkville, Victoria 3050 Australia; 5 School of Public Health and Preventive Medicine, Monash University, Melbourne, Victoria 3004 Australia; 6 Murdoch Children’s Research Institute, Melbourne, VIC 3052 Australia; 7 Department of Pediatrics, University of Melbourne, Royal Children’s Hospital, Melbourne, Victoria 3010 Australia; 8 Department of Neurology, Royal Children’s Hospital, Melbourne, Victoria 3052 Australia; 9 Department of Pediatrics, Peking University People’s Hospital, Beijing, 100000 China; 10 Department of Pediatrics, Peking University First Hospital, Beijing, 100034 China

**Keywords:** Ketogenic diet, Beta-hydroxybutyrate, Reference range, Children, Epilepsy

## Abstract

**Background:**

The ketogenic diet (KD) therapy is a primary treatment for drug-resistant epilepsy, and beta-hydroxybutyrate (BHB) is the main ketone produced during KD. However, the pattern of increase in BHB levels is not well understood, and the reference range for BHB need to be defined. The aim of this study was to evaluate the BHB levels in the first three months, especially one week, after KD initiation, and to explore the physiological reference range for BHB.

**Methods:**

In our study, a fasting initiation strategy was used for the majority of patients (252/300, 84%) who underwent fasting for 24–48 h, the rest fasted for at least 12 h. The concentration of blood BHB was measured four times a day during the first week, at one month and three months. Seizure frequency was recorded at one week, one month and three months. Responders were defined as those with a seizure reduction 50% or more compared to baseline. BHB levels were compared between responders and non-responders. The BHB levels of responders were used to calculate the reference range.

**Results:**

A total of 300 patients were recruited, of whom 172 (57%) had accessible BHB data. BHB levels rapidly rose to 2.0 mmol/L at 19 h, peaked at 4.2 mmol/L at 43 h of therapy, and stabilized by three months. The reference range for BHB was 1.1 to 4.9 mmol/L.

**Conclusions:**

BHB levels increased rapidly following fasting, reaching the peak at day 2, stabilizing from the end of the first week through three months. The lower reference limit for BHB to ensure KD efficacy should be set at 1.1 mmol/L.

**Supplementary Information:**

The online version contains supplementary material available at 10.1186/s42494-024-00199-8.

## Background

The classical ketogenic diet (KD) has been used for the treatment of epilepsy in children for more than 100 years [1, 2]. Despite its demonstrated efficacy [3–5], individual responses to the KD vary significantly because patients with epilepsy have heterogeneous etiologies, and the KD has multiple mechanisms of action. Animal studies have shown that both the KD and exogenous ketones have anti-seizure effects which act in a dose-dependent manner [6–8]. In clinical practice, there is currently no biomarker available to monitor KD efficacy in patients. This lack of a therapeutic target (physiological level) makes it challenging to evaluate whether the KD is effective or adequate in treating patients with epilepsy.

The KD changes brain metabolism from glucose to ketone bodies as the main source of energy. Ketone bodies include beta-hydroxybutyrate (BHB, 78%), acetoacetate (ACA, 22%) and acetone (traces). Although previous studies have suggested that blood BHB correlates with the anti-seizure effect of the KD after one month from initiation [9], or at three and six months [10, 11], randomized controlled clinical trials have suggested a correlation between blood BHB levels and the anti-seizure effect of KD at six weeks [12] or at three months [4]. However, the reference range of BHB for effective seizure control has not been established [3, 13]. Furthermore, understanding the changes in BHB levels during the initiation of KD therapy and throughout the treatment period will assist in correlation with anti-seizure effects of the KD [14–17]. Therefore, we aimed to investigate the temporal changes in blood BHB and derive a reference value of blood BHB concentration associated with early treatment response in children with drug-resistant epilepsy.

## Methods

### Subjects

This was a retrospective cohort study of patients with drug-resistant epilepsy treated with KD therapy, the clinical trial registration number was ChiCTR-IIR-16008342. The study included children with drug-resistant epilepsy who were admitted to Shenzhen Children’s Hospital between June 2014 and November 2017 to commence the KD. Inclusion criteria were as follows: (1) aged 2 months to 25 years; and (2) with drug-resistant epilepsy, defined as failure of treatment with two or more appropriately selected antiseizure medications (ASMs) [18]. Exclusion criteria included: (1) metabolic disorders related to fatty acids metabolism, such as deficiencies in β-oxidative, carnitine, or pyruvate carboxylase; (2) other severe disorders associated with liver or kidney dysfunction, including renal calculi, hyperlipidemia, and immunodeficiency disorders; and (3) lack of parental adherence to the KD.

Written informed consent was obtained from the adult patients or from the parents or legal guardians of minors and individuals with intellectual disabilities. Families were educated about potential efficacy and possible adverse effects of the KD. The study was approved by the Ethics Committee of Shenzhen Children’s Hospital (2023061).

### Methods

#### Initiation of the KD

Baseline seizure frequency was recorded in the patient’s seizure diaries prior to the initiation of the KD. At the time of KD initiation, patients were hospitalized (279/300, 93%) for an average of 6 days. The majority of patients (252/300, 84%) underwent fasting for 24–48 h, the rest fasted for at least 12 h, until they met one of the following criteria: blood BHB concentration ≥ 2.5 mmol/L, urinary ketone (acetoacetic acid) > 3 + , or a fasting period of 48 h [19, 20]. Each patient then commenced the ketogenic formula (Qitong, Zeneca, Shenzhen) at a ratio of 2: 1 to 4: 1 (ratio of fat to carbohydrate plus protein by weight), with a total calorie intake of 60–80 kcal/kg/day. KD foods were gradually introduced, providing one-third of the total calorie intake on the first day, two-thirds on the second day, and the full calorie intake on the third day. Daily supplements, including multivitamins and trace elements with minimal carbohydrate content, were commenced. In addition, potassium citrate was administered to prevent renal calculi, and L-carnitine was prescribed if the patient was deficient in free carnitine. Concomitant ASMs remained unchanged during the first 3 months of KD treatment.

#### BHB monitoring and follow-up

The concentrations of blood BHB were measured at time point 7:00, 12:00, 17:00, and 23:00 for the first five to seven days after KD initiation, and at 7:00 AM after one month and three months of KD therapy. A finger prick blood test was taken to measure BHB levels with Beijing Yicheng Biostrips, Sentest type T1. Parents or guardians were trained to measure BHB levels using a portable machine that had been calibrated according to a standard protocol at the hospital.

**Fig. 1:**
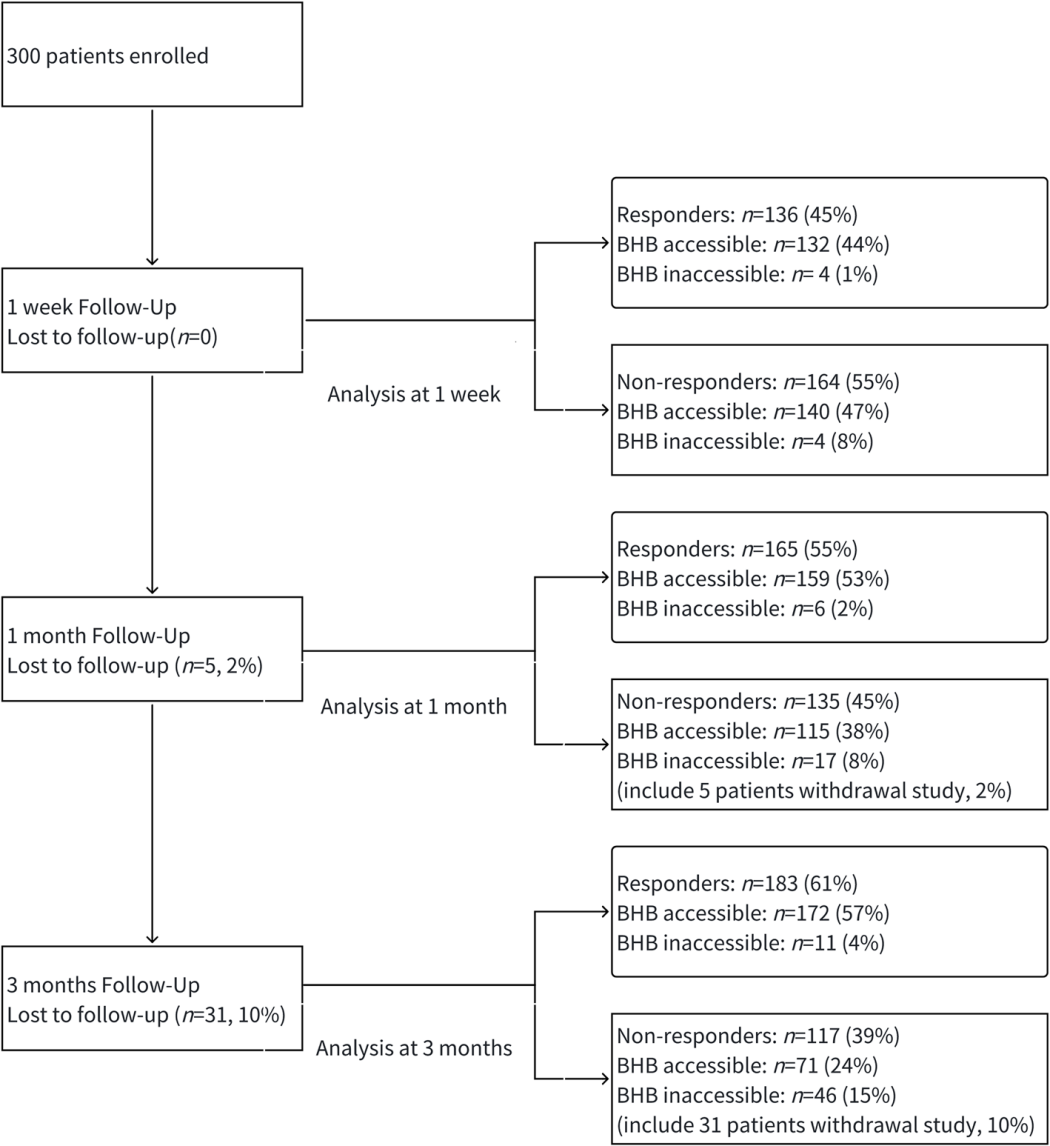
Flow of children through the trial

Regarding KD therapy adherence, no patients withdrew within the first week, 5 patients (2%) withdrew within the first month, and 31 patients (10%) withdrew within three months, as shown in the Fig. 1. At one month, complete BHB records were available for 274/300 (91%) cases, and at three months, complete BHB records were available for 243/300 (81%) cases. Patients were followed up via phone and in clinics by both pediatric neurologists and dietitians. Parents or guardians were instructed to record seizures and any adverse effects following the commencement of KD. Body weight and height were measured weekly.

### Statistical analysis

Seizure frequency was defined as the total number of seizures occurring over a 28-day period. The seizure frequency after 1 month and 3 months of the KD was compared with a baseline period of one month. Response was classified as: (1) non-responders: < 50% seizure reduction, (2) responders: seizure reduction of 50%, or greater. Analysis was performed on an intention-to-treat basis. Data were analysed using SPSS version 26.0 (IBM Corporation, Armonk, NY, USA). The Kolmogorov–Smirnov test was performed to assess normality of quantitative data. For non-normally distributed data, median and interquartile range (IQR) were reported. Qualitative data were statistically described by frequency and percentage. The chi-square test was used to compare qualitative variables between different groups. The median concentration and IQR of BHB at different time points were calculated. Differences in BHB concentrations between time points and efficacy were compared using repeated measures of ANOVA, paired samples *t*-test, and independent samples *t*-test. The reference range of BHB (medical reference = mean ± 1.96SD) was derived from the BHB concentrations (normal distribution) in children treated with KD whose seizure reduction ≥ 50% after three months.

## Results

### Cohort and responders

The study included 300 children whose clinical features are summarized in Table 1, including 191 males (64%) and 109 females (36%). The age of epilepsy onset ranged from newborn to 14 years old, with a median of 8 months (IQR 4,26). The initiation age of ketogenic diet ranged from 2 months to 25 years old, with a median of 32 months (IQR 14,66). The duration from seizure onset to KD initiation was 14 months (IQR 6,34). The number of ASMs (including those discontinued) were 4 (IQR 3,5). Most patients 252 (84%) began treatment with fasting.

**Table 1:** Baseline demographic characteristics of patients

	Total	R	N	Test statistic	*P* value
	300	*n* = 183	*n* = 117		
Gender					
Male *n* (%)	191 (64)	110 (60)	81 (69)	χ^2^ = 2.567	*P* = 0.109
Female *n* (%)	109 (36)	73 (40)	36 (31)		
Age of epilepsy onset (month) m (IQR)	8 (4, 26)	8 (4, 26)	7 (4, 24)	U = 10,090	*P* = 0.445
Age of KD initiation (month) m (IQR)	32 (14, 66)	35 (15, 66)	29 (13,59)	U = 10,050	*P* = 0.371
Seizures or Syndrome *n* (%)				χ^2^ = 0.664	*P* = 0.739
Focal epilepsy	113 (38)	65 (36)	48 (41)		
Generalized seizures	8 (3)	5 (3)	3 (3)		
Syndromes					
Angelman syndrome	2 (1)	2 (1)	0 (0)		
Autism	3 (1)	1 (1)	2 (2)		
Autoimmune encephalitis	12 (4)	10 (5)	2 (2)		
Dravet syndrome	8 (3)	6 (3)	2 (2)		
FIRES	1 (1)	0 (0)	1 (1)		
LGS syndrome	7 (2)	6 (3)	1 (1)		
Mitochondrial diseases	3 (1)	3 (2)	0 (0)		
Ohtahara syndrome	10 (3)	7 (4)	3 (3)		
TSC with epilepsy	6 (2)	4 (2)	2 (2)		
West syndrome	122 (41)	69 (38)	53 (45)		
Unknown	5 (2)	5 (3)	0 (0)		
Total ASM failed *n* (IQR)	4 (3,5)	4 (3,5)	4 (4,5)	U = 11,708.5	*P* = 0.046^*^
Fasting initiation *n* (%)	252 (84)	158 (86)	94 (80)	χ^2^ = 1.910	*P* = 0.167

*R* responders, *N* non-responders, *KD* ketogenic diet, *FIRES* febrile infection related epilepsy syndrome, *LGS* Lennox-Gastaut syndrome, *TSC* tuberous sclerosis complex, *ASM* antiseizure mediations, *BMI* body mass index; m, median

Univariate analysis Chi-Square test (χ2), Mann–Whitney U test

^*^level of significance *P* < 0.05

The types of seizures or syndrome included 113 patients (38%) with focal seizures, 8 patients (3%) with generalized seizures; 122 patients (41%) with infantile epileptic spasms syndrome (West syndrome), 12 patients (4%) with infection or immune-related encephalitis, 10 patients (3%) with Ohtahara syndrome, 8 patients with Dravet syndrome (3%), 7 patients with Lennox-Gastaut syndrome (2%), 6 patients with TSC combined with epilepsy (2%), 3 patients with epilepsy combined with autism (1%), 3 patients with mitochondrial disease combined with epilepsy (1%), 2 patients with Angelman syndrome (1%), febrile infection-related epilepsy syndrome (FIRES) was found in 1 case (1%), and the type of seizures was unknown in 5 cases (2%) (see Table 1). Except the number of anti-seizure medications, there were no differences in baseline characteristics between responders and non-responders. Of the 300 children, 183 (61%) cases had a ≥ 50% seizure reduction (responders) after 3 months of the KD treatment. The response to KD improved over time, with 165/300 (55%) and 136 (45%) classified as responders at one month and one week, respectively.

### Changes of BHB levels with duration of KD therapy

**Fig. 2:**
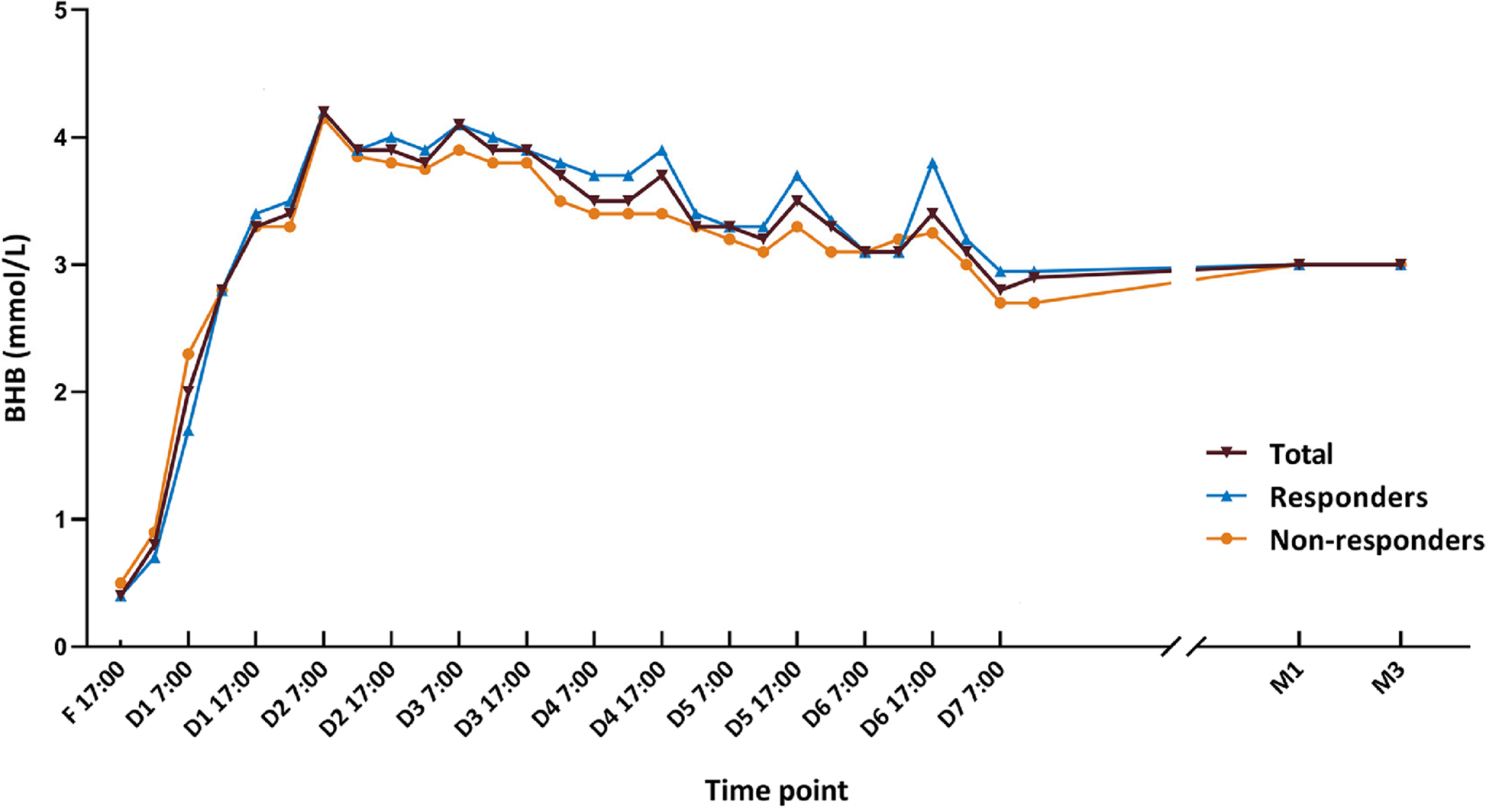
Median (BHB) levels at different time points. BHB, beta-hydroxybutyrate; F, fasting day; D, day; M, month

After fasting commenced, we found that the level of BHB increased rapidly to a relatively high median level of 2.0 mmol/L (IQR 0.8, 3.7) at 19 h (*n* = 300), and further increasing to 2.8 mmol /L (IQR 1.6, 3.8) after 24 h (*n* = 270). By the end of the first week on the KD, the BHB level began to stabilize (Fig. 2 and Table S1).

**Fig. 3:**
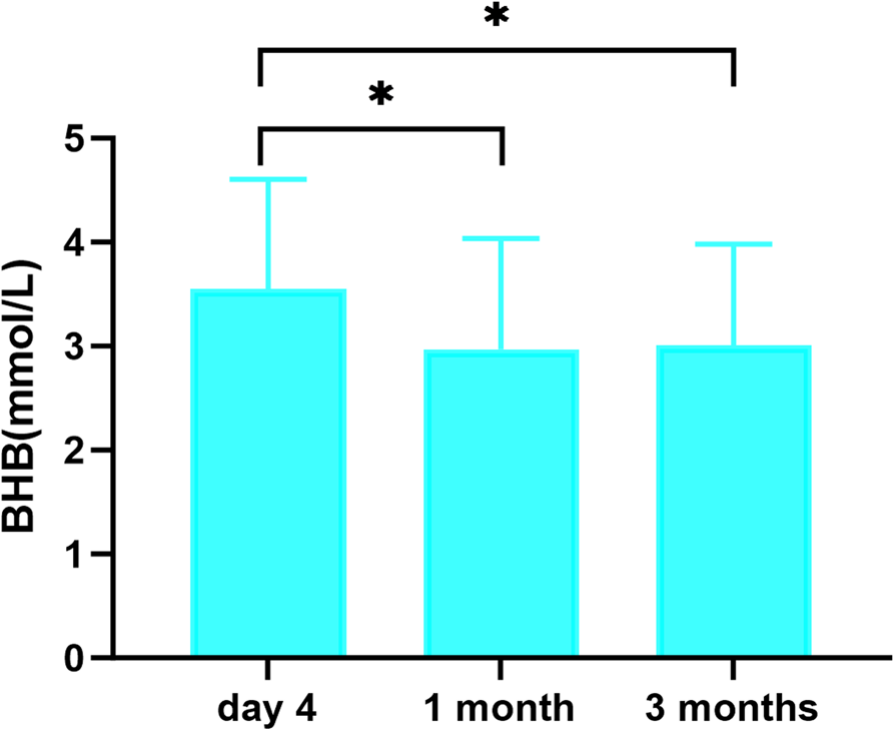
BHB levels compared with day 4, 1 month and 3 months. * means *P* < 0.001

We analysed changes in the BHB profile over three months. In the first week, we observed the highest number of cases on day 4, at which point the BHB level had stabilized, so we used this data to compare the BHB levels at one month and three months of KD treatment. Repeated measures of ANOVA (225 cases, 75%) and Wilcoxon singed-rank test were performed to analyse BHB levels. Comparing day 4 with one month, the BHB concentration decreased by 0.60 mmol/L (95% confidence interval [CI] 0.47–0.71, *P* < 0.001). Similarly, the concentration decreased between day 4 and three months by 0.55 mmol/L (95% CI 0.50–0.60, *P* < 0.001). There was no significant difference in blood BHB level between one month and three months (*P* = 1.00, see Figure 3). Thus, BHB levels had stabilized by one month of KD therapy.

In responders, BHB reached a stable level of 2.8 mmol/L (IQR 1.4, 3.8) at 24 h (12:00, day 1) (*n* = 165) and a peak value of 4.2 mmol/L (IQR 3.3,4.9) at 43 h (7:00, day 2) (*n* = 164). The mean blood BHB level at one month (*n* = 175) was 2.97 ± 1.07 mmol/L (median 3.0, IQR 2.2–3.7, medical reference 0.9–5.0). The reference BHB level in responders at three months (*n* = 172) was 3.01 ± 0.97 mmol/L (median 3.0, IQR 2.3–3.8, medical reference 1.1–4.9). The paired *t*-test was performed to compare BHB levels, and the difference between one month and three months was not statistically significant (*P* = 0.70).

### Correlation of BHB level with KD responses

The total number of responders at one week, one month and three months after KD initiation were 136 (45%), 165 (55%) and 183 (61%), respectively (*P* < 0.001). In the responder group, we analyzed whether a specific BHB level correlated with responder status. However, we did not find a significant relationship between BHB level and KD response at one week, one month or three months (Table 2). The blood BHB level at three months did not differ between responders (3.01 ± 0.97 mmol/L, *n* = 172) and non-responders (3.04 ± 0.92 mmol/L, *n* = 71), *P* = 0.84.

**Table 2:** Median BHB level in responders and non-responders

BHB level (mmol/L)	1 week		1 month		3 months	
	R	N	R	N	R	N
	132	140	159	115	172	71
≤ 2	10 (8%)	14 (10%)	33 (21%)	29 (25%)	33 (19%)	10 (14%)
> 2 and ≤ 4	72 (55%)	87 (62%)	98 (62%)	75 (65%)	115 (67%)	52 (73%)
> 4	50 (38%)	39 (28%)	28 (18%)	11 (10%)	24 (14%)	9 (13%)
	χ^2^ = 3.209, *P* = 0.201		χ^2^ = 3.757,* P* = 0.153		χ^2^ = 1.097, *P* = 0.578	

*BHB* β-hydroxybutyrate, *R* Responder, *N* Non-responders

## Discussion

We report BHB levels in a large cohort of 300 patients at the initiation of the classical KD over the first three months of therapy. We compared BHB levels from the first week with those from the first and third months, defining a reference range for BHB in individuals on the KD. Our findings demonstrate that BHB levels climb rapidly, peaking by day 2, followed by a slight decrease, and then remain stable from one month to three months of KD therapy. Therefore, BHB levels may provide an ideal means to monitor the early implementation of ketosis and definitively indicate that patients have achieved ketosis.

We suggest that clinicians can choose different strategies to start KD according to the patient's condition. For patients with low BHB level, the KD should be continued if seizures are controlled; this strategy is suitable for chronic epilepsy patients undergoing long-term treatment of KD. If seizures are not controlled, the BHB level can be increased to 4.0–6.0 mmol/L for further observation. Conversely, for patients requiring rapid disease control, a high BHB level at the start may be more appropriate. There is increasing pressure to implement the KD in outpatient, due to demands on inpatient services around the world. In outpatient settings, the rapid escalation of ketosis is less important. However, there has been considerable interest in rapid initiation of the KD, particularly with reports of its efficacy in status epilepticus, such as febrile-infection related syndrome [21] and super-refractory status epilepticus [22, 23]. In the intensive care unit, there is a strong emphasis on achieving ketosis as quickly as possible, ideally within one day. Using our fasting protocol, BHB levels reached above 2.0 mmol/L in 19 h, showing rapid establishment of ketosis.

A key metabolic outcome of the KD is the production of ketone bodies, which exhibit various antiseizure effects. Ketones affect both inhibitory and excitatory neuronal transmission, including gamma-aminobutyric acid (GABA), purine, ATP-sensitive potassium channels, and vesicular glutamate transporters. What’s more, ketones also influence mitochondrial function, epigenetic regulation and exhibit anti-inflammatory functions [24].

Our finding of a peak BHB level at day 2 following KD initiation differs from some previous studies. In the 2009 study of 145 children by Neal et al. [4], an increse in BHB levels was found after the three months of diet therapy. However, BHB levels were only measured at baseline and 3 months of the therapy, so an early peak potentially had been missed [4, 25]. Similarly, Buchhalter et al. measured BHB levels at baseline and one month in 23 patients, suggesting that they may have missed both the earlier peak and the rapid achievement of high ketone levels within a few days of KD initiation [26]. A study conducted by Bergqvist and colleagues compared fast and slow KD initiation in 48 children, aligned with our findings [27]. Their group of 24 children with fast initiation showed a similar BHB profile to our cohort, stabilizing by day 2–4 of the therapy. Another study, Anastasia Dressler and colleagues described ketosis in infants (aged < 1 year) on KD with or without the inclusion of breast milk. They found both groups achieved relevant ketosis (BHB ≥ 2 mmol/L) within 41–47 h.Specifically, the BHB level at day 2 was 3.1 mmol/L (IQR 0.5, 4.9) within breast milk group and 3.8 mmol/L (IQR 2.2, 4.9) in standard KD group [28], which were slightly lower than ours results.

Using our cohort of 300 patients, we defined a reference range for blood BHB levels. For the responders, defined as those whose epileptic seizures were reduced by 50% or more at three months, BHB levels in 172 patients were used to calculate the effective reference range. At three months, the BHB level showed a normal distribution with a mean of 3.01 ± 0.97 mmol/L (mean ± SD, IQR 2.3, 3.8; *n* = 172). Therefore, the reference range for therapeutic BHB concentration was established as 1.1–4.9 mmol/L.

In 1976, Huttenlocher [9] suggested that the effect of the KD depends on maintaining a blood BHB level above 2.0 mmol/L. European guidelines for infants with refractory epilepsy for KD recommended BHB levels should not exceed 5.0 mmol/L [16], leading to the currently accepted range of 2.0–5.0 mmol/L. Our findings indicate that 24 (13%) of our responders have BHB levels below 2.0 mmol/L at three months. This suggests that the reference range should be modified. We propose that the lower level of the target BHB level for KD efficacy should be adjust to 1.1 mmol/L. Maintaining lower BHB levels would allow patients to adopt a more relaxed approach to KD treatment, providing a wider variety and quantity of food options. This flexibility can improve patients’ compliance, reduce the burden on caregivers, and enable family members to share ketogenic meals together, thereby enhancing their confidence in managing the disease through dietary therapy.

The blood BHB concentration did not significantly differ between responders and non-responders. This finding is analogous to the levels of other ASMs. For example, therapeutic carbamazepine levels can be ascertained in both responders and non-responders, and these levels do not necessarily reflect the efficacy of medicine [29].

Our study has several limitations. First, it is a single-center study using retrospective data. Additionally, the children were not randomized into the specific BHB concentration ranges, which raises the possibility that those with more difficult-to-treat epilepsy may have been placed on a higher ratio diet and stricter diet regimen to increase BHB levels. This situation is analogous to phase IV clinical trials, which generally show wide ranges of drug levels among responders and non-responders, with no significant difference between groups [27]. Furthermore, all but 29 of our cohort of 300 patients underwent fasting. Although many centers no longer use fasting as a protocol for initiating the KD, fasting can be easily implemented in emergency situations, and enables a more rapid establishment of ketosis in time-critical settings.

## Conclusions

In conclusion, this study indicates that blood ketone body BHB reached maximal levels two days after fasting initiation of the KD and stabilized thereafter. The established BHB reference range at three months was 1.1 mmol/L to 4.9 mmol/L. These data will inform urgent implementation of the KD in cases of status epilepticus and will help define a reference range for BHB levels related to KD efficacy in future studies.

## Supplementary information

Additional file 1: Table S1.

## Data Availability

The datasets of current study are available from the corresponding author on reasonable request.

## Abbreviations

ACA: Acetoacetate
ASMs: Antiseizure medications
BHB: Beta-hydroxybutyrate
GABA: Gamma-aminobutyric acid
KD: Ketogenic diet
IQR: Interquartile range
