# Supplementary material for: Exploring physiological beta-hydroxybutyrate level in children treated with the classical ketogenic diet for drug-resistant epilepsy
Source: Acta Epileptol. 2025 Feb 7;7:10. doi: 10.1186/s42494-024-00199-8 (PMC11960278; doi:10.1186/s42494-024-00199-8)
Supplement: Supplementary file 1 — Additional file 1: Table S1. [file 42494_2024_199_MOESM1_ESM.docx]

| **Supplementary Table 1 Median BHB level from fasting to day 7 (mmol/L), median (IQR)**  n  12:00 BHB  n  17:00 BHB  n  23:00 BHB  n | | | | | | | | | |
| --- | --- | --- | --- | --- | --- | --- | --- | --- | --- |
|  |  | 7:00 BHB | n | 12:00 BHB | n | 17:00 BHB | n | 23:00 BHB | n |
| Start | T 300 | / | / | Fasting begins | / | 0.4(0.2,1.0) | 200 | 0.8(0.3,1.6) | 241 |
|  | **R 183** | / | / | Fasting begins | / | **0.4(0.1,1.0)** | **122** | **0.7(0.3,1.5)** | **152** |
|  | N 117 | / | / | Fasting begins | / | 0.5(0.2,1.3) | 78 | 0.9(0.4,1.9) | 89 |
| Day 1 | T 300 | 2.0(0.8,3.7) | 264 | 2.8(1.6,3.8) | 270 | 3.3(2.4,4.4) | 272 | 3.4(2.6,4.2) | 269 |
|  | **R 183** | **1.7(0.7,3.6)** | **159** | **2.8(1.4,3.8)** | **165** | **3.4(2.4,4.4)** | **163** | **3.5(2.7,4.2)** | **165** |
|  | N 117 | 2.3(0.9,4.0) | 105 | 2.8(1.8,3.8) | 105 | 3.3(2.5,4.3) | 109 | 3.3(2.5,4.2) | 104 |
| Day 2 | T 300 | 4.2(3.1,4.9) | 273 | 3.9(3.1,4.6) | 272 | 3.9(3.1,4.5) | 266 | 3.8(2.9,4.4) | 263 |
|  | **R 183** | **4.2(3.3,4.9)** | **165** | **3.9(3.1,4.6)** | **168** | **4.0(3.1,4.6)** | **164** | **3.9(2.9,4.5)** | **164** |
|  | N 117 | 4.2(2.9,4.8) | 108 | 3.9(3.0,4.6) | 104 | 3.8(3.0,4.4) | 102 | 3.8(3.0,4.4) | 99 |
| Day 3 | T 300 | 4.1(3.1,4.8) | 276 | 3.9(3.0,4.5) | 270 | 3.9(3.0,4.5) | 266 | 3.7(3.0,4.5) | 265 |
|  | **R 183** | **4.1(3.2,4.9)** | **168** | **4.0(3.0,4.7)** | **165** | **3.9(3.2,4.6)** | **160** | **3.8(3.0,4.5)** | **161** |
|  | N 117 | 3.9(3.1,4.6) | 108 | 3.8(2.9,4.4) | 105 | 3.8(2.9,4.4) | 106 | 3.5(2.9,4.4) | 104 |
| Day 4 | T 300 | 3.5(2.9,4.5) | 267 | 3.5(2.7,4.3) | 255 | 3.7(2.9,4.3) | 249 | 3.3(2.8,4.2) | 247 |
|  | **R 183** | **3.7(2.8,4.7)** | **161** | **3.7(2.7,4.3)** | **154** | **3.9(2.8,4.5)** | **149** | **3.4(2.8,4.3)** | **149** |
|  | N 117 | 3.4(2.9,4.3) | 106 | 3.4(2.9,4.2) | 101 | 3.4(3.0,4.1) | 100 | 3.3(2.7,4.0) | 98 |
| Day 5 | T 300 | 3.3(2.5,4.2) | 245 | 3.2(2.6,4.0) | 200 | 3.5(2.9,4.2) | 201 | 3.3(2.5,4.0) | 195 |
|  | **E 183** | **3.3(2.5,4.3)** | **149** | **3.3(2.6,4.1)** | **121** | **3.7(2.9,4.3)** | **123** | **3.4(2.6,4.1)** | **118** |
|  | I 117 | 3.2(2.4,4.0) | 96 | 3.1(2.6,3.8) | 79 | 3.3(2.9,4.2) | 78 | 3.1(2.5,3.9) | 77 |
| Day 6 | T 300 | 3.1(2.4,3.9) | 184 | 3.1(2.2,3.9) | 99 | 3.4(2.5,4.3) | 95 | 3.1(2.4,3.9) | 86 |
|  | **R 183** | **3.1(2.5,4.0)** | **111** | **3.1(2.2,4.1)** | **62** | **3.8(2.4,4.3)** | **57** | **3.2(2.3,4.0)** | **53** |
|  | N 117 | 3.1(2.3,3.7) | 73 | 3.2(2.1,3.7) | 37 | 3.3(2.6,4.1) | 38 | 3.0(2.7,3.7) | 33 |
| Day 7 | T 300 | 2.8(2.3,3.9) | 79 | 2.9(2.3,3.5) | 49 | / | / | / | / |
|  | **R 183** | **3.0(2.4,3.8)** | **52** | **3.0(2.3,3.7)** | **34** | / | / | / | / |
|  | N 117 | 2.7(2.3,4.0) | 27 | 2.7(2.3,3.4) | 15 | / | / | / | / |

“T” total; “R” responders; “N” non-responders; “BHB” beta-hydroxybutyrate; “/” not applicable.
